# Multisensory benefits for speech recognition in noisy environments

**DOI:** 10.3389/fnins.2022.1031424

**Published:** 2022-10-20

**Authors:** Yonghee Oh, Meg Schwalm, Nicole Kalpin

**Affiliations:** ^1^Department of Otolaryngology-Head and Neck Surgery and Communicative Disorders, University of Louisville, Louisville, KY, United States; ^2^Department of Speech, Language, and Hearing Sciences, University of Florida, Gainesville, FL, United States

**Keywords:** multisensory speech recognition, visual, vibrotactile, temporal coherence, speech-in-noise

## Abstract

A series of our previous studies explored the use of an abstract visual representation of the amplitude envelope cues from target sentences to benefit speech perception in complex listening environments. The purpose of this study was to expand this auditory-visual speech perception to the tactile domain. Twenty adults participated in speech recognition measurements in four different sensory modalities (AO, auditory-only; AV, auditory-visual; AT, auditory-tactile; AVT, auditory-visual-tactile). The target sentences were fixed at 65 dB sound pressure level and embedded within a simultaneous speech-shaped noise masker of varying degrees of signal-to-noise ratios (−7, −5, −3, −1, and 1 dB SNR). The amplitudes of both abstract visual and vibrotactile stimuli were temporally synchronized with the target speech envelope for comparison. Average results showed that adding temporally-synchronized multimodal cues to the auditory signal did provide significant improvements in word recognition performance across all three multimodal stimulus conditions (AV, AT, and AVT), especially at the lower SNR levels of −7, −5, and −3 dB for both male (8–20% improvement) and female (5–25% improvement) talkers. The greatest improvement in word recognition performance (15–19% improvement for males and 14–25% improvement for females) was observed when both visual and tactile cues were integrated (AVT). Another interesting finding in this study is that temporally synchronized abstract visual and vibrotactile stimuli additively stack in their influence on speech recognition performance. Our findings suggest that a multisensory integration process in speech perception requires salient temporal cues to enhance speech recognition ability in noisy environments.

## Introduction

Speech perception is essential for both normal-hearing (NH) and hearing-impaired (HI) listeners to communicate effectively in their everyday lives. Because listeners are often in complex listening environments with a lot of background noise and competing voices, accurate speech perception can often be very difficult, especially for HI listeners. Assistive listening devices (ALDs), such as remote microphone systems and noise-canceling headphones are frequently recommended and used to help listeners in these difficult listening situations. In addition to using ALDs, listeners can use other sensory cues to enhance degraded auditory signals and, thus, improve their speech perception in noisy environments. In recent years, several studies have been conducted to investigate the effectiveness of integrating other sensory cues, specifically visual and tactile cues, to enhance listeners’ speech perception in these difficult listening situations. Many of these studies have shown that multisensory integration can enhance a listener’s speech perception abilities from the auditory-only condition: auditory-visual (AV: Sumby and Pollack, 1954; Grant and Seitz, 2000; Grant, 2001; Bernstein et al., 2004; Yuan et al., 2020, 2021a,2021b), auditory-tactile (AT: Gick et al., 2008; Cieśla et al., 2019), and auditory-visual-tactile (AVT: Derrick et al., 2019) conditions.

### Benefits from visual cues

Previous studies that focused solely on the bimodal auditory-visual (AV) relationship and how it can affect listeners’ speech perception in noisy environments have shown that adding a visual cue to enhance a degraded auditory signal can provide speech intelligibility benefits for listeners (Sumby and Pollack, 1954; Grant and Seitz, 2000; Grant, 2001; Bernstein et al., 2004). Most of the previous studies showed that the visual articulation cues from a speaker’s lip movements can provide speech perception benefits. Based on this finding, using these visual articulation cues can be very helpful for listeners in situations where they are able to view the speaker’s lip movements. However, this isn’t always possible. In situations where the speaker’s articulation cues are not visible (i.e., when wearing a mask or turned away from the listener), the listener cannot utilize the articulation cues. To find a solution for listeners in these situations, Yuan et al. (2020) explored the use of amplitude envelope information that’s been extracted from target speech signals as non-articulatory visual cues and found that this information does enhance listeners’ speech perception in noise. This was an important finding because it showed that listeners’ speech perception performance could be enhanced using visual cues other than lip movements, which are not always available. While this study provided investigators with the evidence that using amplitude envelope information as a visual cue can benefit speech perception in noise, it also raised another question: Are there specific modulation parameters that should be applied when extracting amplitude envelope information that will provide the most AV benefits for listeners’ speech perception in noise?

In a follow-up study to their original study, Yuan et al. (2021a) investigated this, focusing specifically on amplitude modulation rate and amplitude modulation depth. They found that there are optimal modulation parameters that provide more AV benefits for speech perception in noise when used than others. Based on their findings, the optimal amplitude modulation rate to use when extracting the amplitude envelope information from the target speech signal is 10 Hz and the optimal amplitude modulation depth is 75%. Overall, these studies have provided evidence that non-articulatory visual cues, specifically amplitude envelope information, can be used to benefit listeners’ speech perception in noise when articulatory cues aren’t available and that certain amplitude modulation parameters can provide more AV speech perception benefits for listeners than others.

### Benefits from tactile cues

Other studies have explored the bimodal auditory-tactile (AT) relationship and how the addition of a tactile cue to an auditory signal in the presence of background noise can benefit listeners’ speech perception. In their study, Gick et al. (2008) investigated the interactions between auditory-tactile and visual-tactile cues and whether these interactions enhanced speech perception for listeners in noise. Their findings revealed that tactile information can enhance speech perception performance in background noise by about 10% compared to the auditory-only (AO) condition in untrained NH listeners, regardless of which additional sensory modality was being used (auditory or visual). However, this study used disyllables (i.e., /aka/, /aba/, etc.) rather than full sentences or words for the auditory cue and the Tadoma method, which involves listeners placing their hands in specific positions around the speaker’s mouth (Gick et al., 2008), to provide the tactile cue rather than a vibrotactile device. In a later study, Cieśla et al. (2019) assessed the benefit of tactile stimulation on listeners’ speech recognition performance in a more realistic listening environment by using Hearing in Noise Test (HINT) sentences as the auditory cue and a sensory substitution device (SSD) that transformed low-frequency speech signals into vibrations delivered to the participants’ fingertips as the tactile cue. Their findings showed that participants’ speech recognition performance improved by about 6 dB in the AT condition in comparison to the AO condition, which implies that the integration of tactile cues can be beneficial for NH listeners in difficult listening environments.

### Benefits from both visual and tactile cues

In addition to the bimodal AV and AT relationships, the tri-modal relationship between all three sensory cues (auditory, visual, and tactile) and how it can benefit listeners’ speech perception in noise has also been investigated. Derrick et al. (2019) investigated this relationship and found that visual and tactile stimuli “stack” together when they’re temporally synchronized with the auditory signal and can enhance the speech perception of NH listeners in noise. Furthermore, they found that while the visual and tactile stimuli presented together had the strongest influence on speech perception performance, visual stimuli alone had a stronger influence on speech perception than tactile stimuli did alone and that adding either visual, tactile, or a combination of the two led to stronger speech perception performance in noise in comparison to the AO condition. From this study, we can assume that multisensory integration can improve listeners’ overall speech perception in noise, however, this study primarily assessed speech detection of syllables (i.e., /ga/vs. /pa/) in noise, used articulatory cues as the visual stimulus (i.e., a speaker’s lip movements) and aero-tactile information as the tactile stimulus (i.e., puffs of air coordinated with the target speech signal that was applied to the listeners’ suprasternal notch), and did not investigate how this tri-modal relationship could benefit HI listeners’ speech perception in noise.

The aim of the current study is to examine the benefits of multisensory integration using either visual and/or tactile cues for sentence recognition in varying levels of background noise. Aside from previous studies on the benefits of multisensory integration for speech perception at the syllable or word level, this study examines the benefits of visual and tactile cues for speech recognition at the sentence level by adding either dynamic visual or tactile cues that are temporally synchronized with a target speech signal to determine to what extent NH listeners utilize these cues in noisy environments. A sphere-shaped visual analog that’s synchronized with the amplitude envelope information of the target speech signal, rather than lip movements, was used as the visual cue in this study. Furthermore, a vibrotactile device taped to participants’ wrists that was also synchronized with the amplitude envelope information of the target speech signal, rather than an aero-tactile device, was used as the tactile cue in this study. This study had four conditions: auditory-only (AO), auditory-visual (AV), auditory-tactile (AT), and auditory-visual-tactile (AVT). We hypothesized that multisensory integration across all conditions (AV, AT, and AVT) will provide speech recognition benefits in complex listening environments, and will provide the most benefit in the AVT condition. If the multisensory cues used in this study are able to elicit improvement of speech-in-noise performance in NH individuals, an interesting path to possibly elicit speech-in-noise enhancement for HI individuals, who often show poorer speech perception ability in noisy listening situations than in NH listeners, is revealed.

## Materials and methods

### Subjects

Twenty young adult subjects (18 females; 20–24 years old) participated in the experiment. All subjects had normal audiometric hearing thresholds (air conduction thresholds ≤ 25 dB hearing level), and were screened for normal cognitive function using a Mini-Mental State Examination with a minimum score of 27 out of 30 required to qualify (MMSE; Folstein et al., 1975; Souza et al., 2007). All subjects had normal or corrected-to-normal vision, and none of the subjects reported any impairment in somatosensory processing. All subjects were paid an hourly wage and completed all experiments in between one to two sessions of 1 h each. No prior experience with psychophysical research was required for participation; however, practice tutorials (10 minutes) were provided to all subjects in order to assure familiarity with the procedures. All experiments were conducted in accordance with the guideline for the protection of human subjects as established by the Institutional Review Board (IRB) of the University of Florida, and the methods employed were approved by that IRB.

### Stimulus materials

For the audio stimuli, two different speech materials were used as target sentences and a simultaneous speech-shaped noise (SSN) masker in our experiment. The target speech material was an open set that consisted of 500 Harvard sentences (IEEE, 1969). Of the 500 sentences, 250 were spoken by a male native English speaker, while the other 250 were spoken by a female native English speaker. Twenty sentences were chosen for the practice section, and 480 sentences were used for the actual experiments: 120 sentences for the audio-only (AO) condition, 120 sentences for the audio-visual (AV) condition; 120 sentences for the audio-tactile (AT) condition; 120 sentences for the audio-visual-tactile (AVT) condition. One example of a sentence utilized is,

“*Steam hissed from the broken valve.*”

The embedded SSN masker has the same spectrum as the long-term averaged Harvard sentence spectrum. The target stimuli were sampled at 44,100 Hz and root-mean-square (RMS) matched through MATLAB at a fixed 65 dB sound pressure level (SPL) for presentation. All target sentences were embedded within a SSN masker of various degrees of target signal to noise masker ratios; SNRs: −7, −5, −3, −1, and 1 dB. For example, the −7 dB SNR indicates that the maker level is 7 dB louder than the target level (i.e., masker: 72 dB SPL; target: 65 dB SPL).

**FIGURE 1:**
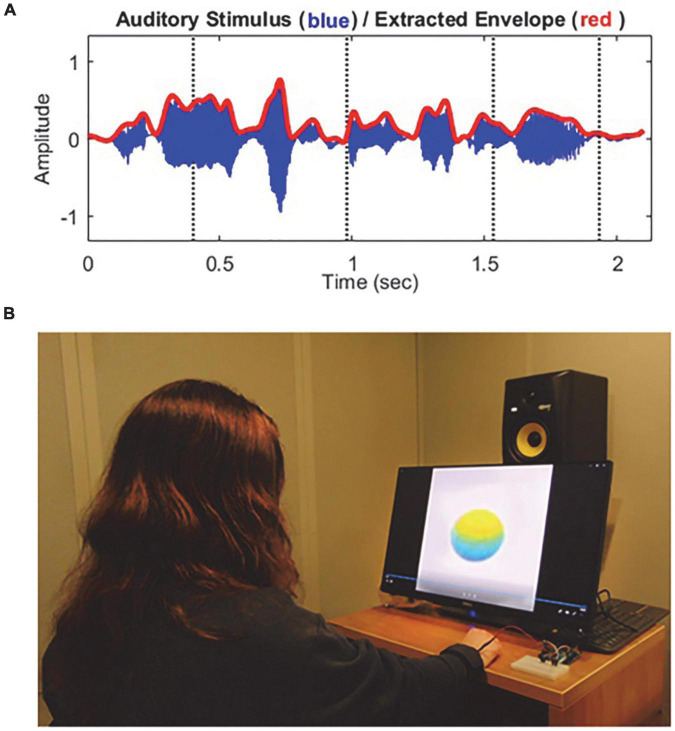
Schematic representation of the stimuli **(A)** and experimental configuration **(B)** used in this study. **(A)** The blue thin line indicates the waveform of a sample target sentence “Steam hissed from the broken valve,” and the red thick line indicates the extracted amplitude envelope which was used for the volume size of the visual stimulus (sphere-shaped abstract ball) and strength of the tactile stimulus (vibration). **(B)** Auditory stimuli were presented *via* a loudspeaker, visual stimuli were displayed on a 24-in monitor, and vibrotactile stimuli were delivered *via* a DC vibration motor to the subject’s right wrist using a wristband.

For both visual and tactile stimuli, the amplitude envelope for each target sentence was extracted for generating multimodal stimuli. Figure 1A shows the waveform (blue) and the extracted temporal envelope (red) of a sample speech sentence “Steam hissed from the broken valve.” The visual stimuli consisted of a colored sphere that changed volume with each syllable in the target speech sentence. The sphere changed volume in temporal synchrony with the acoustic amplitude envelope of each target sentence. Here, the speech envelope was extracted using the Hilbert function in MATLAB (version R2019a, MathWorks, Natick, MA, USA), and low-pass filtered with a cutoff frequency of 10 Hz (i.e., 10-Hz amplitude modulation rate) with a fixed modulation depth at 75%. Please find more detailed information about visual stimulus generation in our previous studies (Yuan et al., 2020, 2021a,2021b). Similar to the visual stimulation, the extracted speech envelope was transferred through vibrotactile stimulation. That is, the vibrotactile stimulus strength was synchronized with the amplitude of the target speech envelope, and stimulation was delivered with the pulse width modulation (PWM) technique (Barr, 2001). Here, the vibrotactile stimulation has been shown to be an effective means of delivering tactile information to humans in previous studies (e.g., Bark et al., 2014).

### Procedure

The experiment was conducted in a single-walled, sound-attenuating booth. All stimuli were generated using custom MATLAB (version R2019a, MathWorks, MA, USA) scripts. Auditory stimuli were routed through an RME Babyface Pro audio interface (RME Audio, Haimhausen, Germany) and delivered *via* a frequency-equalized loudspeaker (Rokit 5, KRK Systems, FL, USA), positioned in the front hemifield a distance of 1 m from the center of the listener’s head. The output of the loudspeaker was calibrated using a Brüel and Kjaer sound level meter with an A-weighting filter (Brüel and Kjaer Sound and Vibration Measurement A/S, Naerum, Denmark). Visual stimuli were displayed on a 24-in touch screen monitor (P2418HT, Dell, TX, USA), positioned below the loudspeaker. Vibrotactile stimuli were processed through an ARDUINO microcontroller board (UNO Rev3, Arduino LLC, MA, USA) with a haptic motor controller (DRV2605L, Adafruit, New York, USA) and delivered *via* a DC vibration motor (DC 3 V 85 mA 12,000 rpm Flat Button-type Motor, DZS Electronics, TX, USA) to the right wrists of participants using a wristband. Figure 1B shows an example experimental configuration for auditory, visual, and tactile stimuli used in this study.

Four hundred eighty sentences were presented to the subject: 120 sentences for the AO condition, and 120 sentences for each multimodal stimulus condition (AV, AT, and AVT) with an SSN noise. For each stimulus condition, half of the sentences were male talkers and another half were female talkers, and subjects were tested with those different talker gender conditions in separate sessions. It is noted that previous studies reported that the target talker’s gender could affect speech recognition performance in multi-talker listening situations (e.g., Oh et al., 2021). In the current study, the male and female voices were used as one factor to explain this gender-specific difference in multisensory speech perception benefits. Each of the SNR levels (−7, −5, −3, −1, and 1 dB) was repeated 12 times per level, resulting in five SNR blocks and a total of 60 target sentences. The presentation order of different SNR blocks was randomized for each subject. Target sentences with different conditions were also randomly presented within each block. Subjects were asked to pay attention to the target sentence until the stimulus was fully presented and then repeat what they heard. Each target sentence was only presented once. Data scoring was calculated based on complete sentences. The percentage of words accurately identified in each sentence was calculated and cross-checked by two trained research assistants in the lab. All of the scoring processes were based on our project-scoring guide. For instance, if a word is missing a phoneme, or has a typo but is still clearly the same word (like photograph vs. photography), it was scored as correct.

## Results

**FIGURE 2:**
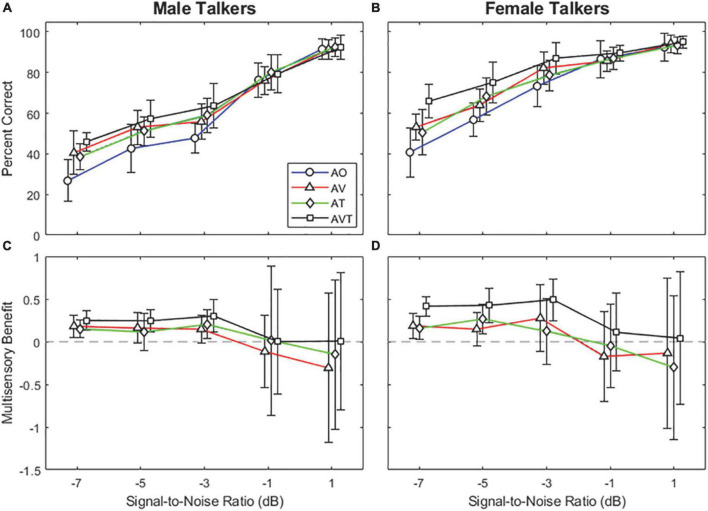
Average word recognition accuracy results as a function of various SNR levels for male talker **(A)** and female talker **(B)** conditions. Average multisensory benefits as a function of various SNR levels male talker **(C)** and female talker **(D)** conditions. Error bars represent standard deviations of the mean. The horizontal dashed lines in the lower panels indicate the reference (zero) benefit.

Figures 2A,B show the average word recognition accuracy as a function of various SNR levels for the male and female talkers, respectively. For all stimulus conditions (AO, AV, AT, and AVT), average subject responses demonstrate an S-shaped perceptual curve (i.e., psychometric curve). For the male talker (Figure 2A), approximately 8–20% improvement of the mean word recognition accuracy was observed in the lower three SNR levels between −7 and −3 dB, when synchronized multimodal cues were provided. A similar trend was also observed for the female talker (Figure 2B): approximately 5–25% improvement between −7 and −3 dB of SNR. In general, greater word recognition improvement was observed in the AVT stimulus condition for both talker genders (male talker: 15–19%; female talker: 14–25%), compared to either AV or AT stimulus condition. The greatest improvement was observed at the lowest SNR level of −7 dB (AO to AVT: 27–46% for the male talker and 41–66% for the female talker).

A linear mixed model (LMM) analysis was used to analyze the data with the word recognition accuracy as a dependent variable, the talker gender (male vs. female), the stimulus condition (AO, AV, AT, and AVT), and the SNR (−7, −5, −3, −1, and 1 dB) as fixed factors, and the subject as a random factor. The model specification was as follows: Accuracy ∼ 1 + Gender + Stimulation + SNR + Gender: Stimulation + Gender: SNR + Stimulation: SNR + Gender: Stimulation: SNR + (1| Subject). The LMM results showed main effects of all three fixed factors [gender: *F*_(1,741)_ = 694, *p* < 0.001; stimulation: *F*_(3,741)_ = 94.7, *p* < 0.001; SNR: *F*_(4,741)_ = 1189.4, *p* < 0.001] and a significant interaction between gender and SNR [*F*_(4,741)_ = 54.8, *p* < 0.001] and between stimulation and SNR [*F*_(12,741)_ = 11.8, *p* < 0.001], however, no interaction between gender and stimulation (*p* = 0.08) was observed.

*Post-hoc* pairwise comparisons using Bonferroni correction were computed to better understand the word recognition accuracy among the stimulus conditions (i.e., AO vs. AV, AO vs. AT, AO vs. AVT) in the lower three SNRs (−7, −5, and −3 dB). The results demonstrated that the word recognition accuracy was significantly improved with the addition of combined visual and tactile cues (i.e., AVT) in all three SNR conditions for both talker genders (*p* < 0.001 for all cases). For the visual or tactile stimulus condition (AV or AT), significant differences were only observed in the lowest SNR (−7 dB) level (*p* < 0.006 for all cases). Please see the Supplementary Table 1 for other pairwise comparison results. The higher two SNRs (−1 and 1 dB) were counted as ceiling effects. In order to demonstrate the relative amount of gain from integrating an auditory and visual (and/or tactile) cue, we applied the formula for multisensory benefits scores, which was introduced in the audiovisual integration study by Sumby and Pollack (1954):


(1) M⁢u⁢l⁢t⁢i⁢s⁢e⁢n⁢s⁢o⁢r⁢y⁢B⁢e⁢n⁢e⁢f⁢i⁢t=(A⁢V⁢(o⁢r⁢A⁢T⁢o⁢r⁢A⁢V⁢T)-A⁢O)/(100-A⁢O)

Figures 2C,D display the multisensory benefit curves as a function of SNR levels. It should be noted that the multisensory benefit formula applied in this study (Equation 1) has limitations in representing the data showing the ceiling effect (Ross et al., 2007). Thus, the benefits scores are limited to explaining the multisensory benefit, especially calculated at the highest SNR condition. The results show that mean multisensory benefits were positive and similar at the low three SNR levels (−7, −5, and −3 dB). For the male talker (Figure 2C), the benefit scores were approximately 0.16 for AV and AT conditions and 0.28 for the AVT condition. Interestingly, the benefit score based on combined visual and tactile cues (AVT) was roughly the sum of that in the visual cue (AV) and that in the tactile cue (AT). A similar but more distinct trend was observed for the female talker (Figure 2D). The benefit scores were approximately 0.2 for AV and AT conditions and 0.44 for the AVT condition.

## Discussion

The present study explored the benefits of multisensory integration for speech recognition in noisy environments for NH listeners. The results show that the addition of temporally-synchronized visual (and/or tactile) cues to a noise-degraded speech signal did provide improvements in speech recognition performance, especially at the lower SNR levels of −7, −5, and −3 dB (male talker: 8–20% improvement; female talker: 5–25% improvement). Word recognition accuracy was shown to have improved the most with the addition of both visual and tactile cues (i.e., see the square symbols in Figure 2 for the AVT condition) at all three SNR levels for both gender talkers. In other words, while some benefits were observed in the conditions where either a visual or tactile cue was added (i.e., see the triangle and diamond symbols in Figure 2 for the AV and AT conditions, respectively), the greatest improvement in word recognition performance (15–19% improvement for males and 14–25% improvement for females) was observed in the AVT condition when both visual and tactile cues were temporally integrated. Additionally, significant improvements in performance in the AV and AT conditions were only observed at the lowest SNR level of −7 dB. The results also show that the mean multisensory benefit scores for the AVT condition are approximately equal to the combined scores of the AV and AT conditions for the lower three SNR levels (−7, −5, and −3 dB).

Similar multimodal benefits were observed in many previous studies in the auditory-visual domain (Sumby and Pollack, 1954; Grant and Seitz, 2000; Grant, 2001; Bernstein et al., 2004). One of the first studies to examine the benefits of utilizing visual cues on speech perception performance was conducted by Sumby and Pollack (1954). Their study found a maximum of 80% improvement in participants’ speech recognition performance in noise, especially in the lowest SNR condition (−30 dB), when articulatory visual cues were provided. This improvement in speech recognition performance in an auditory-visual (AV) condition is greater than the amount of benefit found in the present study, which may be explained by the different methodologies used in Sumby and Pollack’s work. Their study used single-word utterances embedded in background noise, rather than full sentences, and gave participants a closed set of words to choose from when asked about what they heard. Thus, in terms of listening effort, our study required more from participants, which may have contributed to the difference in benefits between the studies. Additionally, their study used articulatory visual cues (i.e., target speaker’s lip movements) rather than a temporally-synchronized envelope cue as the visual cue, which could also explain the difference in our findings.

Grant and Seitz (2000) explored the benefits of articulatory visual cues further in their study, and their findings suggest that visual cues taken from facial movements during speech production do enhance speech detection when the movements are temporally-aligned (i.e., “matched”) with the acoustic envelopes of the speech signals. Specifically, when the facial movements matched the target sentence, an improvement in speech detection thresholds of approximately 1.6 dB was observed. Another study by Bernstein et al. (2004) also found that articulatory visual cues could enhance speech detection performance across participants. The group mean detection threshold amongst participants was lowest in the auditory-visual condition (−18 dB SNR) and increased (−16 dB SNR) when only the auditory signal was provided. The results from the Grant and Seitz (2000) and Bernstein et al. (2004) studies suggest that adding a visual cue to the target auditory signal can provide benefit in noise, however, their studies focused on speech detection thresholds (i.e., the ability to determine whether a speech signal is present or absent) in noise, not speech recognition performance (i.e., the ability to determine if a speech signal is present and repeat the utterance back), which is what the present study aimed to focus on. Furthermore, both of these studies involved participants listening to monosyllabic utterances, rather than full sentences. These various experimental conditions could explain the differences in the multisensory benefits obtained between previous studies and the current study.

In the auditory-tactile domain, previous studies that aimed to determine the benefit of adding tactile cues have also shown an improvement in speech perception performance (Gick et al., 2008; Cieśla et al., 2019). For example, Cieśla et al.’s (2019) study found that the integration of tactile cues can benefit speech perception performance in noisy environments when the tactile cue was provided through a Sensory Substitution Device (SSD), which converted the auditory signal into a tactile vibration that was then delivered to the index and middle fingers on each participant’s dominant hand. Their study used recorded HINT sentences for the target signal, which were each embedded in varying levels of a six-talker babble to create a more difficult listening environment. They found about a 4% improvement in speech recognition performance when comparing the AO condition (mean performance score of 21.25%) to the AT condition (mean performance score of 25%). Our study did find a larger improvement (8−20% for male gender talker; 5−25% for female gender talker) perhaps due to differences between the placement and programming of the vibrotactile device as well as the sentences used. Unlike Cieśla et al. (2019), our vibrotactile device was attached to the subject’s right wrist, a placement that may be more sensitive to changes in vibration. We also used the temporal speech envelope as the vibrotactile cue, whereas Cieśla and colleagues only focused on the portions of speech involving lower frequencies. Lastly, we used Harvard sentences instead of HINT sentences.

Interestingly, the integration of both visual and tactile cues has been shown to improve speech perception performance. In their 2019 study, Derrick et al. found that the addition of visual and tactile cues allows for benefits to “stack” together when integrated together with audio than when either cue is integrated individually (AV-AO; 17 dB or AT-AO; 2 dB). The authors identified the mean SNR level at which the signal was correctly identified with 82% accuracy. It should be noted that the Derrick et al. (2019) study primarily assessed the speech detection of syllables (i.e., /ga/vs. /pa/) in speech noise, used articulatory cues as the visual stimulus (i.e., a speaker’s lip movements) and aero-tactile information as the tactile stimulus (i.e., puffs of air coordinated with the target speech signal that was applied to the listeners’ suprasternal notch). The benefits measured by Derrick et al. (2019) may have been higher due to the use of only two monosyllabic syllables, which are similar to the findings in other previous studies mentioned in the auditory-visual and the auditory-tactile domains. The authors themselves mentioned that most subjects experienced a ceiling effect, which means, the subjects were still able to guess the correct sound presented by using the visual information provided despite overpowering the speech by over 30 dB. Our study used more complex speech samples (Harvard sentences), thereby making the testing more difficult than Derrick et al.’s (2019). By using the sentences to test using multisensory modalities, our testing was able to replicate more realistic listening situations as subjects are not likely to experience listening to monosyllabic speech in noise.

A series of recent audiovisual integration studies by Oh and his colleagues (Yuan et al., 2020, 2021a,2021b) also found significant sentence recognition improvements by using an abstract visual cue rather than an articulatory visual cue (i.e., lip movement) that many of the aforementioned studies used. The current study expanded upon their findings in order to determine whether similar benefits would be observed in the tactile domain. Using the same modulation parameters that the aforementioned studies found to provide the most speech recognition benefit (10 Hz modulation rate and a 75% modulation depth) for our abstract multisensory cues in each of our conditions (AV, AT, and AVT), our study not only found similar benefits in the tactile domain but also that these abstract visual and tactile cues additively “stacked” together to provide even greater benefit in the AVT condition. Specifically, when provided together (AVT), the visual and tactile cues provided double the amount of benefit in both the male-talker (15–19%) and female-talker (14–25%) conditions than when provided alone in the AV and AT conditions.

Previous studies have investigated the potential mechanisms that may underlie this speech-in-noise perception benefit through the addition of multisensory cues, specifically with the addition of visual cues. One potential mechanism, bimodal coherence masking protection (BCMP), was proposed by Grant and Seitz (2000). Their study suggests that articulatory visual cues (i.e., lip movements or facial movements) can add additional information about the spectral and temporal characteristics of the target speech signal and can provide speech perception benefits in noisy environments. In other words, adding additional energy (i.e., articulatory visual cues) within a separate spectral region than the target speech signal in noise can release the target signal from the noise and, thus, can provide speech perception benefits. In our study, the abstract visual and vibrotactile cues provided the same amplitude envelope information as the target speech signal, thus this cross-modal temporal coherence might have enhanced the target signal perception and consequently improve participants’ speech-in-noise perception performance. However, the current study has a limit to explaining speech perception benefits possibly from cross-modal spectral coherence. Further studies should explore the interaction between spectral and temporal coherences together across all three sensory domains.

Another possible mechanism for the results found in the current study could be related to attentional cues. The review studies by both Koelewijn et al. (2010) and Hillyard et al. (2016) suggested that multisensory integration, especially in the auditory-visual domain, and involuntary attentional process could occur interdependently, and this integration and attentional cueing interaction could be observed in the cortical regions related to each sensory input. Regarding our findings in this study, the temporally coherent multisensory cues may have improved the attention of the subject to the auditory input, thereby improving their ability to perceive the speech target in noisy environments. More studies will be needed to better understand the mechanism underlying interaction between perceptual integration across three different sensory inputs and related attentional processes. This could shed light on how to use multisensory cues for speech perception enhancement in noisy environments.

For this study, some limitations apply. Any results from this study can be limited due to a listener’s age or gender bias since the majority of our participants were young, college-aged females. This can lead to difficulties in generalizing our study’s findings to the general population. In particular, the results in the current study showed a difference in participants’ performance based on the gender of the target speaker. This difference in performance between male and female talkers was not due to a difference in the amplitude envelope cue, instead, it might be due to the participant’s gender bias. Thus, more controlled studies need to explain the talker-specific (and/or listener-specific) differences in multisensory benefit. Furthermore, our participants were only allowed a 10-min training session to become familiar with the procedures of the study. Perhaps with more practice and time, the benefit scores may be more significant. For instance, if the participants had become more familiar with the abstract visual and vibrotactile stimuli presented, they would be able to use them more optimally. Additionally, the current study used the same modulation parameters (i.e., 10 Hz modulation rate and 75% modulation depth) as found in our previous study (Yuan et al., 2021a) for all three of our multimodal conditions (AV, AT, and AVT). While these parameters were found to be optimal in the visual domain, we did not examine whether these parameters were the most optimal for the tactile domain. Thus, listeners’ multimodal benefits in speech-in-noise performance may vary with different modulation rates and depths for the vibrotactile cue. Future studies should aim to find the most optimal modulation parameters for the auditory-tactile domain. Lastly, the current study did not include an asynchronized (i.e., “mismatched”) multimodal stimulus condition. In other words, a condition where the temporal cue was not matched with the sensory modality being provided. A condition in which the temporal cue is no longer synchronized with the sensory modality being added could lead to a decrease in speech perception performance or potentially improve performance even more, so it’s important for future studies to explore this asynchronized temporal integration as well.

## Conclusion

To our knowledge, this is the first study that explored dynamic abstract visual and vibrotactile cues as multisensory inputs in speech-in-noise performance at the sentence level. The findings in this study show that multisensory interactions can be fundamentally important for speech perception ability in normal-hearing listeners. Multisensory speech perception in auditory, visual, and tactile domains requires salient temporal cues to enhance speech recognition ability in noisy environments. The amplitude envelope, serving as a reliable temporal cue source, can be applied through different sensory modalities (i.e., visual and/or tactile) when auditory ability is compromised by various background noises. Further testing should include listeners with hearing loss to determine how the benefits of multisensory integration for speech perception in noise for this population compare to that of NH listeners. The findings may be applied to future rehabilitation approaches using auditory training programs to enhance speech perception in noise, and implications for potential technological enhancements to speech perception with hearing devices—in particular, the integration of a non-acoustic signal.

## Data availability statement

The original contributions presented in this study are included in the article/Supplementary material, further inquiries can be directed to the corresponding author.

## Ethics statement

The studies involving human participants were reviewed and approved by the Institutional Review Board (IRB) of the University of Florida. The patients/participants provided their written informed consent to participate in this study.

## Author contributions

YO designed the experiments and analyzed the data. All authors performed the experiments, contributed to the article, discussed the results at all states, and approved the submitted version.
